# Evaluation of the quality of guidelines for the management of reduced fetal movements in UK maternity units

**DOI:** 10.1186/s12884-015-0484-5

**Published:** 2015-03-05

**Authors:** Stephanie Jokhan, Melissa K Whitworth, Felicity Jones, Ashleigh Saunders, Alexander E P Heazell

**Affiliations:** Maternal and Fetal Health Research Centre, Institute of Human Development, University of Manchester, Oxford Road, Manchester, UK; St. Mary’s Hospital, Central Manchester University Hospitals NHS Foundation Trust, Manchester Academic Health Science Centre, Manchester, M13 9WL, UK

**Keywords:** Reduced fetal movements, Management, Stillbirth, Stillbirth prevention, Risk management

## Abstract

**Background:**

The development of evidence-based guidelines is a key step in ensuring that maternity care is of a universally high standard. To influence patient care national and international guidelines need to be interpreted and implemented locally. In 2011, the Royal College of Obstetricians and Gynaecologists published guidelines for the management of reduced fetal movements (RFM), which can be an important symptom of fetal compromise. Following dissemination it was anticipated that this guidance would be implemented in UK maternity units. This study aimed to assess the quality of local guidelines for the management of RFM in comparison to published national standards.

**Methods:**

Cross-sectional survey of maternity unit guidelines for RFM. The guidelines were assessed using the Appraisal of Guidelines for Research and Evaluation (AGREE) II Tool and scored by two independent investigators. Two national guidelines were used as standards to evaluate unit guidelines.

**Results:**

Responses were received from 98 units (42%); 12 units had no guideline. National guidelines scored highly using the AGREE II tool but there was wide variation in the quality of individual maternity unit guidelines, which were frequently of low quality. No guidelines incorporated all the recommendations from the national guideline. Maternity unit guidelines performed well for clarity and presentation but had low scores for stakeholder involvement, rigour of development and applicability.

**Conclusions:**

In contrast to national evidence based guidance the quality of maternity unit guidelines for RFM is variable and frequently of low quality. To increase quality, guidelines need to include up to date evidence and audit standards which could be taken directly from national evidence-based guidance. Barriers to local implementation and resource implications need to be taken into consideration. Training may also improve the implementation of the guideline. Research is needed to inform strategies to realize the benefits of clinical guidance in practice.

## Background

The presence of fetal movements (FM) is a well-recognised sign of fetal viability and vitality [1]. In contrast to the reassurance provided by normal fetal activity, reduced fetal movements (RFM) can be an important symptom preceding fetal death; this was initially recognised in cohorts of women completing fetal movement charts [2]. These findings are confirmed by recent studies, O’Sullivan et al. describe a 2–3 fold increased risk of subsequent stillbirth in women who present with a live baby after RFM [3] and Stacey et al. found a two-fold increase in maternal perception of RFM in the two weeks prior to stillbirth (adjusted odds ratio 2.37; 95% CI: 1.29–4.35) [4]. Biomedical studies suggest that maternal perception of RFM is associated with stillbirth by underlying placental dysfunction [5-7]. Thus, it is hypothesised that maternal awareness of fetal movements and standardised management to detect fetal compromise could reduce the incidence of stillbirth. One quality-improvement study gives preliminary evidence that this may be the case [8].

The 8^th^ Confidential Enquiry into Stillbirths and Deaths in Infancy (CESDI) reviewed 422 antepartum stillbirths and found suboptimal care in 45% of cases [9]; more than 10% of which related to altered FMs or RFM, including: changes in FMs not voiced by the mother, healthcare professionals not effectively communicating the importance of reporting a change in FMs or inadequate management of RFM [9]. Inadequate management included failure to act in high risk pregnancies and not undertaking appropriate examinations and investigations. Although the 8^th^ CESDI report dates from 1999, these findings were echoed in a regional Confidential Enquiry of stillbirths in 2010–11 [10].

Two related studies, in the UK and Australia/New Zealand describe a wide variation in obstetric and midwifery practice in the management of RFM [11,12]. This may result from a paucity of evidence identified by two systematic reviews that conclude that formal FM counting using specified alarm-limits cannot be recommended and no specific management plan can be proposed [13,14]. Due to the variation in practice and the frequency of suboptimal care in the management of RFM, several studies concluded that a clinical guideline covering the assessment and management of RFM should be developed.

Clinical guidelines are one means to improve and standardise clinical care; they are defined as “systematically developed statements to assist practitioner and patient decisions about appropriate health care for specific clinical circumstances” [15]. Their role in maternity care is strongly supported by the Royal College of Obstetricians and Gynaecologists (RCOG) who emphasise that “optimal standards of clinical care will be achieved only by following national guidelines and through the quality of staff training and clinical research” [16]. Potential positive impacts of clinical guidelines include: improved quality of clinical decisions, reduced variation in care, support for other quality improvement activities and identification of gaps in research evidence. However, guidelines can potentially lead to harm, research evidence could be lacking or misinterpreted, recommendations can be influenced by the guideline developers and inflexible guidelines can prevent care being tailored to individual patients [17]. The RCOG published guidelines for the management of RFM in 2011 [18], it is hoped that good quality guidance would reduce variation in care, increase access to appropriate investigations and reduce perinatal mortality.

This study aimed to systematically assess the quality of guidelines for the management of RFM in individual maternity units and whether this equates with the standard of national guidelines. To ensure robust and reproducible assessment of guidelines the Appraisal of Guidelines for Research and Evaluation (AGREE) II tool was used to evaluate all guidelines [19].

## Methods

Two hundred and thirty five maternity units across the UK were asked by letter for either an electronic or paper copy of their guideline for the management of RFM in May-July 2013. Ethical approval was not required as this work was a service evaluation. In addition, two national evidence-based guidelines were identified by internet searches (Royal College of Obstetricians and Gynaecologists, UK [18]; Australia and New Zealand Stillbirth Alliance (ANZSA) [20]). Unit guidelines were compared against the national evidence-based guidelines. Basic information regarding the guidelines including the authors, review date, version control was recorded as well as the recommendations of the guideline. The recommendations contained within the unit guidelines were compared against twelve recommendations in the RCOG guideline based upon the strongest grade of evidence.

The individual maternity unit and two national guidelines were reviewed and scored by two independent observers (SJ and AH) using the AGREE II tool which has been used previously in maternity care [21,22]. The AGREE II tool has 23 items in 6 domains of Scope and Purpose (3 items), Stakeholder involvement (3 items), Rigour of Development (8 items), Clarity of Presentation (3 items), Applicability (4 items) and Editorial Independence (2 items). The scope and purpose domain assesses whether the aims and target audience are explicitly stated. Stakeholder involvement domain evaluates the involvement of professional users and patients in guideline development. The use of systematic literature searches to identify evidence and the link between evidence and guidance is assessed in the “rigour of development” domain. In addition, this domain assesses whether the guideline has been peer-reviewed and whether a strategy is in place for updating the guideline. Clarity of guidance is assessed in the fourth domain, with an ideal standard of easily identifiable, specific and unambiguous recommendations. The applicability domain assesses whether barriers to implementation and resource implications have been identified, and a strategy to implement guidance into practice has been stated. Finally, editorial independence requires a statement regarding potential conflicts of interests of authors or commissioners. Each item is rated on a seven point Likert scale with 1 being the worst quality and 7 being the best. There is also a global rating score from 1–7. Prior to assessing the guidelines both investigators used AGREE II online training tools to familiarise themselves with the instrument.

Data, including overall scores and domain scores for the guidelines were collated and analysed using Microsoft Excel. The agreement between both appraisers’ (AH and SJ) assessments was compared on 20% of the samples using Cohen’s Kappa coefficient. The relationship between the overall score and the total of the domain scores were calculated by Pearson’s correlation coefficient. Descriptive statistics were used to describe the domain and overall scores; unit guidelines and national guidelines were compared using Mann–Whitney U-test (two-sided comparison; Graphpad version 5, La Jolla, CA). A p value of <0.05 was accepted as statistically significant.

## Results

### Quantitative evaluation of guidelines

Of the 235 units contacted, a response was received from 98 (42%). Of those respondents, 12 (12%) did not have a guideline. Seven (8%) of the 86 guidelines received were out of date and 10 (12%) had no review date. No guideline contained all 12 key recommendations from the RCOG Guideline, the median number of recommendations included in unit guidelines was 7 (Range 3–11; Table 1). The only criterion included in all the guidelines was ‘after fetal viability has been confirmed and history confirms a decrease in fetal movements, arrangements should be made for the woman to have a cardiotocograph (CTG) to exclude fetal compromise’. Eleven units continue to recommend the use of kick-charts despite national guidance to the contrary. Further detailed investigation of women presenting with RFM was recommended in up to 30% of guidelines, which increased to 53% for women with multiple presentations with RFM. There was no relationship between the strength of evidence for the recommendation and inclusion in unit guidelines.

**Table 1:** **Percentage of unit guidelines stating recommendations in RCOG national guideline**

**Recommendation**	**Percentage of guidelines**
Take a clinical history (including risk factors for stillbirth) [B]	79
Clinical examination (including symphysis-fundal height) [B]	81
Auscultation of the fetal heart (with Pinnard stethoscope or handheld Doppler device) [B]	69
Screening for preeclampsia by urinalysis and blood pressure [GPP]	43
After fetal viability has been confirmed and history confirms a RFM, arrangements should be made for the woman to have a cardiotocograph (CTG) to exclude fetal compromise [B]	100
Duration of CTG recording (for at least 20 minutes if over 28w gestation) [B]	13
Ultrasound scan for fetal biometry and umbilical artery Doppler if clinically deemed to be at risk of stillbirth (within 24 h) [B]	30
Ultrasound scan for fetal morphology (if not already done) [A]	7
A selective role for fetal biophysical profile [B]	11
Ultrasound scan for fetal biometry and umbilical artery Doppler in all women with recurrent presentation [B]	53
Women should be reassured that 70% of pregnancies with a single episode of RFM are uncomplicated [C]	46
Avoid the use of kick charts (formal fetal movement counting) [A]	77

Grade of evidence in RCOG guideline is shown in square brackets: [A] At least one high-quality meta-analysis, systematic review or randomised controlled trial with very low risk of bias; [B] A body of evidence including high-quality systematic reviews of case-control or cohort studies or high-quality case–control or cohort studies with a very low risk of confounding, bias or chance and a high probability that are applicable to the target population, and demonstrating overall consistency of results; [C] A body of evidence including well-conducted case–control or cohort studies with a low risk of confounding, bias or chance, directly applicable to the target population and demonstrating overall consistency of results or extrapolated evidence from high-quality systematic reviews, or case-control or cohort studies; *GPP* = Good Practice Point [18].

### Semi-qualitative assessment using the AGREE II tool

Scores obtained by the two independent investigators using the AGREE II tool showed good agreement for the overall score (Κ = 0.7) but more variable results for the individual domains ranging from fair (K = 0.25) to very good (K = 1.00). For both investigators, the Pearson’s correlation coefficient between the total domain scores and overall score was 0.94 indicating a very strong relationship between the overall assessment and the sum of the individual components.

**Figure 1:**
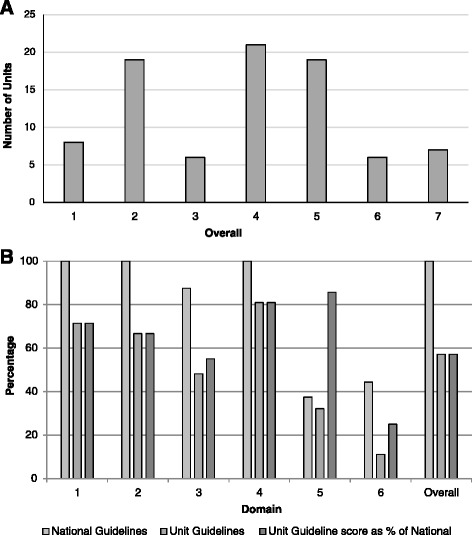
**A) Distribution of overall scores for unit guidelines using AGREE II tool; B) Percentage score for each domain for national and unit guidelines.**

Both the RCOG and ANZSA guidelines received the maximum overall score of 7 and the majority of individual components scored >5 (Table 2). The ANZSA guideline had a low score for editorial independence as it had no statement regarding the provenance of the guideline or competing interests of the authors. The distribution of overall scores of unit guidelines is shown in Figure 1A; scores were not normally distributed, scores of 2 and 5 were most common. The range of individual domain scores for unit guidelines is shown in Table 3. Eight guidelines (9%) with a score of 1 would not be recommended for practice; in these guidelines all domain scores were consistently rated ≤3. In contrast, the 7 guidelines which received the maximum overall score had above average in all of the domains. High scoring guidelines had their highest scores in the ‘scope and purpose’ and ‘rigour of development’ domains. Overall, unit guidelines scored significantly lower in all domains and total score compared to national guidelines (Table 3 and Figure 1B). The least difference was seen in the ‘clarity of presentation’ domain and the greatest difference in the ‘editorial independence’ domain.

**Table 2:** **Scores generated using the AGREE II Tool for national guidelines for the management of reduced fetal movements**

**Domain**	**RCOG**	**ANZSA**	**Median**
1 – Scope and Purpose [21]	21	21	21
2 – Stakeholder involvement [21]	21	21	21
3 – Rigour of development [56]	50	48	49
4 – Clarity of presentation [21]	21	21	21
5 – Applicability [28]	11	10	10.5
6 – Editorial Independence [14]	14	2	8
Total score [161]	138	123	130.5
Overall score [7]	7	7	7

Maximum score for each domain is shown in square brackets.

**Table 3:** **Scores generated using the AGREE II Tool for unit guidelines for the management of reduced fetal movements**

**Domain**	**Median (Range)**	**P value local vs. national guidelines**
1 – Scope and Purpose [21]	15 (3–21)	0.005
2 – Stakeholder involvement [21]	14 (3–21)	0.005
3 – Rigour of development [56]	27 (8–42)	0.0003
4 – Clarity of presentation [21]	17 (9–21)	0.003
5 – Applicability [28]	9 (4–24)	0.62
6 – Editorial Independence [14]	2 (2–2)	0.02
Total score [161]	85 (41–114)	-
Overall score [7]	4 (1–7)	0.02

The range of scores is shown in parentheses. Maximum score for each domain is shown in square brackets.

When assessing individual units scores differed greatly, but some patterns were apparent. In the first domain, ‘scope and purpose’, the majority of scores were above average quality (≥4). Unit guidelines usually specifically stated the objective of the guideline, but lost points due to a lack of description of to whom the guideline is meant to apply. Guidelines that scored poorly in this domain had either omitted the section or there was insufficient detail. Overall, the ‘stakeholder involvement’ domain was of average quality, but there was a wide range of scores (Table 3). High scoring units named at least one obstetrician and one midwife involved in guideline development. However, the majority of guidelines (57%) did not seek the views of service-users.

The ‘rigour of development’ of unit guidelines was poor, with most units having a low score. None of the unit guidelines explicitly stated a strategy to obtain evidence and few referenced relevant research. Critically, 41% of guideline did not reference national evidence-based guidelines in lieu of a novel literature search. Although a review date was usually included, points were frequently lost because there was rarely a procedure for updating the guideline. In contrast, national guidelines stated an explicit strategy for generating evidence and had a comprehensive reference list.

‘Clarity of Presentation’ was the highest scoring domain and was the biggest collective strength of all the guidelines with no difference between national and unit guidelines; almost all units highlighted the key recommendations for practitioners. In contrast, ‘applicability’ was one of the lowest scoring domains, with both national and unit guidelines neither identifying barriers or facilitators to their implementation nor resource implications. Nevertheless, some high-scoring guidelines provided supporting materials to aid dissemination such as patient information leaflets. All unit guidelines scoring the lowest possible score of 2 for ‘editorial independence’, most likely because in the UK healthcare is publicly funded implying that there are no directly competing interests.

## Discussion

To address deficiencies in the care of women presenting with RFM identified by CESDI and national surveys of practice regarding RFM, ANZSA and RCOG produced guidelines released in 2010 and 2011 respectively [18,20]. The failure to translate evidence-based guidance into local maternity unit guidelines represents a missed opportunity to address variation in care and to potentially reduce stillbirths in this high-risk group. Differences between local and national recommendations described here is not an isolated finding in maternity care; Rowe described low-quality of UK guidelines for transfer of women in labour from midwifery-led units [22] and two international studies, a Danish study of methods of anaesthesia for Caesarean section and a review of guidelines developed by the World Health Organisation (WHO) described variation in guideline quality [21,23]. The WHO study found that newer guidelines were of higher quality than older ones [21], which may be relevant to the guidelines for the management of RFM reviewed here as 20% were out of date or had no specified review date. In addition, even when the same guideline is used, different units vary in their adherence to it, resulting in failure to deliver effective treatment [24]. Thus, local factors need to be addressed when implementing any clinical guidance.

The AGREE II tool had good agreement between investigators with comparable Kappa values of 0.3-0.96 to earlier studies [25] and a very strong relationship between scores for individual domains and the score for overall impression. Interestingly, we observed a very similar pattern in guideline quality to Rowe [22]; the scope and purpose and clarity of presentation scored highly, while stakeholder involvement, rigour of development and applicability received lower scores. Rowe’s study also found that none of the unit guidelines scored higher than the minimum score for editorial independence which suggests that this is probably not relevant for individual unit guidelines in a publically funded healthcare system [22].

The area where unit guidelines performed the weakest was the “rigour of development” domain. There was little description of methodology to obtain evidence and a significant minority of units (41%) did not refer to national guidelines positively or negatively. More worryingly, 13% of units directly contradicted the high-grade evidence obtained from systematic reviews and meta-analyses. This raises the question whether following detailed, transparent guideline development the evidence should remain constant and its applicability be adapted to local settings with relevant knowledge of resources and an ability to identify barriers to change. Since maternity units are not mandated to adopt national guidance, they are instead encouraged to adapt it using local stakeholder groups. Development of guidelines is a resource intensive exercise, requiring expertise and service-user involvement, searching and synthesis of the evidence which is then contextualised. Critically, our study showed that stakeholder involvement and applying guidance to practice were amongst the weakest areas of unit guidelines. Therefore, it could be argued that local resources could be much better employed involving service users, assessing barriers to implementation and adapting guidance to their local situation rather than synthesising evidence.

Although guidelines are not intended to mandate clinical practice, they should maintain the quality of care and remain consistent with evidence-based recommendations [26]. Thus guidelines are constrained by the evidence-base; Prusova et al. identified that only 9-12% of recommendations in RCOG guidelines are based on the highest grade evidence (meta-analysis/high-quality randomised trials) [27]. This highlights the need for more high-quality studies in maternity care, and specifically in RFM where only eleven of the twenty-eight recommendations are based upon the grade A or B evidence. However, local guidelines often did not include the recommendations based upon higher-grade evidence. This suggests that individual units are not selecting only the most evidence-based recommendations for inclusion in their local guidelines, but that the weaknesses identified in the rigour of their development means the quality of evidence might not be adequately appraised.

Ultimately, the difference between national and local guidance may explain why evidence from clinical research does not produce the desired improvements in outcome for the wider population. Here, less than 30% of guidelines recommended ultrasound assessment of fetal growth, liquor volume and umbilical artery Doppler after the first presentation with RFM and 53% after recurrent RFM despite evidence that women with recurrent RFM are at increased risk of stillbirth, that these investigations best identify poor pregnancy outcome and that this approach reduces perinatal mortality [3,8,28]. To address this issue, clinical guidance needs to be supplemented with education and training programmes that openly discuss the underpinning evidence so that clinicians can counsel patients accordingly. For example, measurement of symphysis-fundal height (SFH) using customised growth charts has been recommended by national guidance since 2002, but failed to reduce stillbirths until this was incorporated into the Growth Assessment Programme, which supplemented measurement of SFH with education, regular training and specific audit criteria [29]. The AFFIRM study, stepped-wedge cluster randomised controlled trial (NCT01777022) will address whether training, parent and staff education combined with implementation of the RCOG guidelines on the management of RFM will reduce perinatal mortality.

## Conclusion

This evaluation has shown that unit guidelines for the management of a common presentation in maternity care were of variable quality, and were of a significantly lower standard than national guidelines. This may adversely affect care for women presenting with RFM. Unit guidelines need to be improved in specific areas, particularly stakeholder involvement and incorporating the relevant research identified by national guidance and applying those findings to the local situation.

## Abbreviations

AGREE: Appraisal of Guidelines for Research and Evaluation
ANZSA: Australia and New Zealand Stillbirth Alliance
CESDI: Confidential Enquiry into Stillbirths and Deaths in Infancy
CTG: Cardiotocography
FM: Fetal movements
RCOG: Royal College of Obstetricians and Gynaecologists
RFM: Reduced Fetal Movements
WHO: World Health Organisation
